# Supplementary material for: Development of chemokine network inhibitors using combinatorial saturation mutagenesis
Source: Commun Biol. 2025 Apr 3;8:549. doi: 10.1038/s42003-025-07778-6 (PMC11969024; doi:10.1038/s42003-025-07778-6)
Supplement: Supplementary file 4 — Supplementary Data 2 [file 42003_2025_7778_MOESM4_ESM.zip › SupplementaryData3/scripts/xgeo.tar!/./script/analysis/JK/JK_Human stimulated islet_SuppTableChemokinePoolConstruction.docx]

Supplementary Table: Human stimulated islet Chemokine Pool Construction

| Chemokine | Supplier | Code | Concentration [uM] |
| --- | --- | --- | --- |
| CXCL10 | Peprotech | 300-12 | 3.98789 |
| CXCL11 | Peprotech | 300-46 | 1.84811 |
| CXCL9 | Peprotech | 300-26 | 1.2918 |
| CXCL8 | Peprotech | 200-08 | 0.70482 |
| CX3CL1 | Peprotech | 300-31 | 0.66769 |
| CXCL16 | Peprotech | 300-55 | 0.44904 |
| CXCL1 | Peprotech | 300-11 | 0.34731 |
| CCL2 | Peprotech | 300-04 | 0.16514 |
| CXCL2 | Peprotech | 300-39 | 0.12674 |
| CXCL6 | Peprotech | 300-41 | 0.11593 |
| CXCL5 | Peprotech | 300-22 | 0.08012 |
| CCL5 | Peprotech | 300-06 | 0.06409 |
| CCL11 | Peprotech | 300-21 | 0.04682 |
| CCL22 | Peprotech | 300-36A | 0.03749 |
| CCL20 | Peprotech | 300-29A | 0.01826 |
| CCL8 | Peprotech | 300-15 | 0.01775 |
| CXCL3 | Peprotech | 300-40 | 0.01336 |
| CCL19 | Peprotech | 300-29B | 0.01121 |
| CCL28 | Peprotech | 300-57 | 0.00646 |
